# Positron Emission Tomography Imaging of Macrophages in Atherosclerosis with ^18^F-GE-180, a Radiotracer for Translocator Protein (TSPO)

**DOI:** 10.1155/2018/9186902

**Published:** 2018-05-22

**Authors:** Sanna Hellberg, Heidi Liljenbäck, Olli Eskola, Veronique Morisson-Iveson, Matthew Morrison, William Trigg, Pekka Saukko, Seppo Ylä-Herttuala, Juhani Knuuti, Antti Saraste, Anne Roivainen

**Affiliations:** ^1^Turku PET Centre, University of Turku, Kiinamyllynkatu 4-8, 20520 Turku, Finland; ^2^Turku Center for Disease Modeling, University of Turku, Kiinamyllynkatu 10, 20520 Turku, Finland; ^3^GE Healthcare Ltd., White Lion Road, Amersham, Buckinghamshire HP7 9LL, UK; ^4^Department of Pathology and Forensic Medicine, University of Turku, Kiinamyllynkatu 10, 20520 Turku, Finland; ^5^A.I. Virtanen Institute for Molecular Sciences, University of Eastern Finland, Neulaniementie 2, 70210 Kuopio, Finland; ^6^Science Service Center, Kuopio University Hospital, Puijonlaaksontie 2, 70210 Kuopio, Finland; ^7^Turku PET Centre, Turku University Hospital, Kiinamyllynkatu 4-8, 20520 Turku, Finland; ^8^Heart Center, Turku University Hospital, Hämeentie 11, 20520 Turku, Finland

## Abstract

Intraplaque inflammation plays an important role in the progression of atherosclerosis. The 18 kDa translocator protein (TSPO) expression is upregulated in activated macrophages, representing a potential target to identify inflamed atherosclerotic plaques. We preclinically evaluated ^18^F-GE-180, a novel third-generation TSPO radioligand, in a mouse model of atherosclerosis. *Methods*. Nine hypercholesterolemic mice deficient in low density lipoprotein receptor and apolipoprotein B48 (LDLR^−/−^ApoB^100/100^) and six healthy C57BL/6N mice were injected with 10 MBq of ^18^F-GE-180. Specificity of binding was demonstrated in three LDLR^−/−^ApoB^100/100^ mice by injection of nonradioactive reference compound of ^18^F-GE-180 before ^18^F-GE-180. Dynamic 30-minute PET was performed followed by contrast-enhanced CT, and the mice were sacrificed at 60 minutes after injection. Tissue samples were obtained for ex vivo biodistribution measurements, and aortas were cut into serial cryosections for digital autoradiography. The presence of macrophages and TSPO was studied by immunohistochemistry. The ^18^F-GE-180 retention in plaque areas with different macrophage densities and lesion-free vessel wall were compared. *Results*. The LDLR^−/−^ApoB^100/100^ mice showed large, inflamed plaques in the aorta. Autoradiography revealed significantly higher ^18^F-GE-180 retention in macrophage-rich plaque areas than in noninflamed areas (count densities 150 ± 45 PSL/mm^2^ versus 51 ± 12 PSL/mm^2^, *p* < 0.001). Prominent retention in the vessel wall without plaque was also observed (220 ± 41 PSL/mm^2^). Blocking with nonradioactive GE-180 diminished the difference in count densities between macrophage-rich and noninflamed areas in atherosclerotic plaques and lowered the count density in vessel wall without plaque. *Conclusion*. ^18^F-GE-180 shows specific uptake in macrophage-rich areas of atherosclerotic plaques in mice. However, retention in atherosclerotic lesions does not exceed that in lesion-free vessel wall. The third-generation TSPO radioligand ^18^F-GE-180 did not show improved characteristics for imaging atherosclerotic plaque inflammation compared to previously studied TSPO-targeting tracers.

## 1. Introduction

Atherosclerosis is a disease of the vessel wall involving both systemic and local inflammatory processes. Macrophages have a central role in the development of atherosclerosis, from initiation of the inflammatory process to plaque progression and even plaque rupture [1, 2]. Detection of the level of inflammation in atherosclerosis would have high value in assessing disease activity. An imaging method for that purpose would also advance the development of anti-inflammatory drugs for atherosclerosis, since it would allow following up the treatment efficacy.

Positron emission tomography/computed tomography (PET/CT) imaging is a sensitive and quantitative method for detecting biological processes. Glucose analogue 2-deoxy-2-[^18^F]-fluoro-*D*-glucose (^18^F-FDG) PET/CT is a sensitive method for detecting inflammatory activity in vascular wall based on uptake in inflammatory cells [3, 4]. ^18^F-FDG, however, is not specific for inflammation, and therefore, new imaging tracers for a more specific inflammation detection and therapy evaluation are needed. The 18 kDa translocator protein (TSPO), previously known as peripheral benzodiazepine receptor, is an evolutionarily conserved and ubiquitously expressed protein mainly localized in the outer mitochondrial membrane. It is involved in steroid biosynthesis as well as in mitochondrial cholesterol transport. It has been studied both as a potential therapeutic target and as a target for imaging purposes [5–8]. Imaging of TSPO has been studied mainly in neuroinflammatory diseases, since TSPO is highly expressed in activated microglial cells. The prototypic TSPO PET tracer ^11^C-(*R*)-PK11195 and many new-generation tracers have been evaluated for that purpose [9]. Since TSPO is expressed in active macrophages, there has also been attempts to image atherosclerotic inflammation with TSPO-targeting tracers, both in preclinical [10–12] and clinical [13, 14] settings. Imaging with ^11^C-(*R*)-PK11195 is limited by a low target-to-background ratio, and for many second-generation TSPO radioligands, a genetic polymorphism affects their binding [15]. Therefore, novel tracers would be desirable to overcome these limitations.

S-*N*,*N*-diethyl-9-[2-^18^F-fluoroethyl]-5-methoxy-2,3,4,9-tetrahydro-1H-carbazole-4-carboxamide (Flutriciclamide, ^18^F-GE-180) is a novel, third-generation TSPO radioligand with high affinity and specific binding [16–18]. We aimed at evaluating ^18^F-GE-180 in the imaging of atherosclerotic plaque inflammation in a LDLR^−/−^ApoB^100/100^ mouse model. We studied the tissue distribution and retention of the radiotracer by *in vivo* PET/CT imaging and ex vivo biodistribution. Ex vivo autoradiography was performed to assess the tracer retention in plaque areas with different macrophage densities. The specificity of the ^18^F-GE-180 binding was studied in mice with preinjection of excess of nonradioactive reference compound of ^18^F-GE-180 (nonradioactive GE-180). The presence of TSPO and macrophages in aorta was studied by immunohistochemistry.

## 2. Materials and Methods

### 2.1. Animals

All mice were housed in animal facilities of University of Turku Central Animal Laboratory under standard conditions with lights on from 6.00 am to 6.00 pm. The mice had ad libitum access to water and food throughout the study. All studies were conducted with approval from the Lab-Animal Care & Use Committee of the State Provincial Office of Southern Finland (Reference number ESAVI/1583/04.10.03/2012) and in compliance with the European Union directives relating to the conduct of animal experimentation.

Hypercholesterolemic mouse model deficient in the LDL receptor and apoB48 (LDLR^−/−^ApoB^100/100^) was utilized. The lipid profile of these mice resembles human familial hypercholesterolemia, and they develop large, inflamed plaques in the aortas. Nine male LDLR^−/−^ApoB^100/100^ mice were fed with Western-type diet (TD.88137, Harlan Teklad, consisting of 42% of calories from fat and 0.2% from cholesterol, no sodium cholate, Harlan Laboratories, Madison, WI, USA) for 4-5 months, starting at the age of two months. Six male 6-7-month-old C57BL/6N mice fed with regular chow diet were utilized as healthy controls. The details of studied mice are presented in Table 1.

### 2.2. Radiosynthesis of ^18^F-GE-180

^18^F-GE-180 was synthesized according to the previously described protocol [19]. The molar activity was 1800 ± 740 GBq/*µ*mol at the end of synthesis, and the radiochemical purity was >97% in each tracer batch. The nondecay-corrected radiochemical yield was 53% ± 3%.

### 2.3. In Vivo Imaging

The mice were anaesthetized with 2–2.5% isoflurane inhalation and kept on a heating pad. They were first imaged with CT for attenuation correction (Inveon Multimodality PET/CT, Siemens Medical Solutions, Knoxville, TN, USA). After CT, approximately 10 MBq of ^18^F-GE-180 was administered as an intravenous bolus injection via a tail vein cannula, and a 30-minute dynamic PET was started at the same time with the injection. After PET, 100 *µ*l of intravenous contrast agent (eXIA160XL, Binitio Biomedical Inc., Ottawa, ON, Canada) was injected, and a high-resolution CT was performed as described in [20]. The PET images were reconstructed with 2D ordered-subset expectation maximization (OSEM2D) algorithm with two iterations into 2 × 30 s, 4 × 60 s, and 5 × 300 s time frames, and CT images were reconstructed with a Feldkamp-based algorithm. The radioactivity concentration in tissues was analysed by defining regions of interest (ROIs) with Carimas 2.9 program (Turku PET Centre, Turku, Finland). The ROI for blood was placed in the left jugular vein. The results were extracted as mean standardized uptake values (SUV) and were plotted as time-activity curves. Mean SUVs at 20–30 min after injection were utilized in numerical calculations.

### 2.4. Blocking Study

For three LDLR^−/−^ApoB^100/100^, an *in vivo* blocking study was performed to demonstrate the specificity of ^18^F-GE-180. These mice were injected with excess amount (3 mg/kg) of nonradioactive GE-180 (GE Healthcare Ltd., Amersham, United Kingdom) 5 minutes before the injection of radioactive ^18^F-GE-180. Nonradioactive GE-180 was dissolved in 20% ethanol, 20% dimethyl sulfoxide (DMSO), and 60% sterile water, and the injection volume was 2 ml/kg. After that, we followed the same protocol as in the other mice in the study.

### 2.5. Tissue Sampling and Ex Vivo Biodistribution

At 60 min after injection of ^18^F-GE-180, the mice were sacrificed. Under deep isoflurane anesthesia, the blood was collected by cardiac puncture into heparinized tubes and mice were euthanized with cervical dislocation. Samples of blood and various tissues were collected and weighed, and the radioactivity was measured using a gamma counter (Triathler 3″, Hidex, Turku, Finland) cross-calibrated with a dose calibrator (VDC-202, Veenstra Instruments, Joure, Netherlands). Radioactive decay was corrected to the time of injection, and the dose remaining in the injection site was subtracted. The radioactive concentration measured in the tissue samples was expressed as SUV. For the radioactivity measurements, the aorta from ascending aorta to the level of diaphragm was dissected and blood was removed with saline. The aortic roots of mice were preserved in formalin and embedded in paraffin for histological analyses.

### 2.6. Autoradiography of Aortic Cryosections

The distribution of radioactivity in aorta was studied with digital autoradiography in LDLR^−/−^ApoB^100/100^ mice. The saline-rinsed aorta was frozen, and sequential longitudinal sections of 8 and 20 *μ*m were cut with a cryomicrotome at −15°C and thaw-mounted onto microscope slides. The sections were air-dried and opposed to an imaging plate (Fuji Imaging Plate BAS-TR2025, Fuji Photo Film Co., Ltd., Tokyo, Japan). After an exposure time of 16 h, the imaging plates were scanned with a Fuji Analyser BAS-5000 (Fuji Photo Film Co., Ltd., Tokyo, Japan; internal resolution 25 *μ*m).

### 2.7. Histology and Immunohistochemistry

The 20 *μ*m aortic sections were stained with hematoxylin and eosin for morphology. The 8 *µ*m aortic sections were stained with anti-Mac-3 antibody (Clone M3/84, 1:5000, BD Pharmingen, Franklin Lakes, NJ, USA) to detect the macrophages in plaques. For the aortic roots, serial paraffin sections from the level of the aortic sinus were cut and stained with either anti-Mac-3 or anti-TSPO antibody (NBP1-95674, 1 : 10,000, Novus Biologicals, Littleton, CO, USA) or with Movat's pentachrome stain. The detailed methods for immunohistochemistry have been described previously [11]. The plaque burden was quantitated from the Movat's pentachrome stained aortic root sections as intima-to-media ratio (IMR). The quantitation was performed with ImageJ (NIH, Bethesda, MD, USA).

### 2.8. Autoradiography Analyses

The autoradiographs of 8 *µ*m sections were analysed for count densities (photo-stimulated luminescence per unit area, PSL/mm^2^) with Tina 2.1 software (Raytest Isotopenmessgeräte GmbH, Straubenhardt, Germany). The ROIs were defined to plaques and lesion-free vessel wall according to the histology. The ROIs in plaques were divided to three groups (low, intermediate, and high macrophage infiltration) based on the percentage of Mac-3 positive staining in the plaque area measured by ImageJ (NIH, Bethesda, MD, USA). The radioactivity retention in plaque areas with different macrophage densities and lesion-free vessel wall were compared. The background counts were subtracted, and the retention was normalized to the injected radioactivity dose per gram of mouse weight as well as the proportion of tracer decayed during the exposure time [11].

### 2.9. Statistical Analyses

The results are expressed as mean ±SD. Statistical analyses were conducted with IBM SPSS Statistics 23 (IBM Corp., Armonk, NY, USA). *p* values of less than 0.05 were considered statistically significant. The sample size was evaluated by performing power calculation based on the previous study evaluating aortic PET tracer uptake in LDLR^−/−^ApoB^100/100^ and C57BL/6N mice [20]. According to the power calculation, assuming the average tracer uptake (SUV) to be 1.5 ± 0.11, 5 mice per group would be needed to detect 15% difference in the uptake at 90% probability with the alpha value being 0.05. To compare the tissue radioactivity concentrations of LDLR^−/−^ApoB^100/100^ mice to those of C57BL/6N and blocked LDLR^−/−^ApoB^100/100^ mice in the *in vivo* PET and ex vivo biodistribution analyses, ANOVA with Dunnett's post hoc correction was performed. In autoradiography, the count densities in areas with different macrophage percentages and lesion-free vessel wall were analysed by ANOVA and Tukey's correction. Comparisons between LDLR^−/−^ApoB^100/100^ mice and blocked LDLR^−/−^ApoB^100/100^ mice in autoradiography were performed by Student's *t*-test.

## 3. Results

### 3.1. Histology and Immunohistochemistry

**Figure 1:**
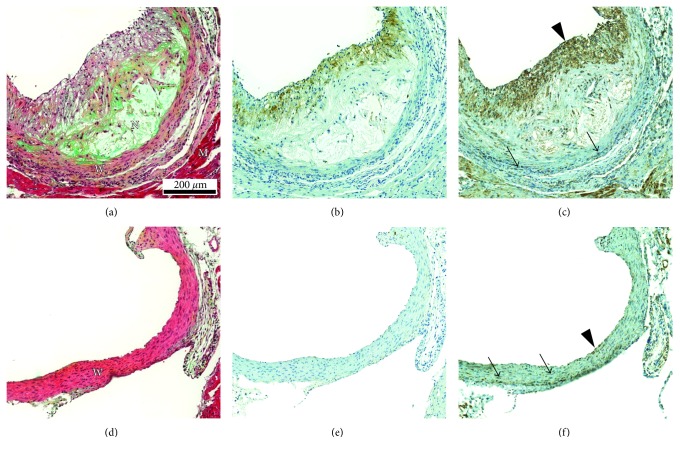
Visualisation of the vascular morphology and the presence of macrophages and TSPO in LDLR^–/–^ApoB^100/100^ and C57BL/6N mice. (a) Movat's pentachrome stained section of LDLR^–/–^ApoB^100/100^ aortic root shows large and cell-rich plaque with necrotic core (N) and fibrous cap (F). W = vessel wall (vascular smooth muscle cells) and M = myocardium. (b) Macrophage immunostaining (Mac-3) of the same plaque. Positive staining (brown colour) is present in the fibrous cap area. (c) TSPO immunostaining. Positive staining (brown colour) is present in the same areas as Mac-3 positive staining and additionally in vascular smooth muscle cells (arrow), endothelial cells (arrowhead), and in the myocardium. (d) Movat's pentachrome stained aortic root section of C57BL/6N mouse shows lesion-free vessel wall lined by endothelium. (e) Mac-3 staining is negative. (f) Positive TSPO staining is seen in both smooth muscle cells (arrows) and endothelial cells (arrowhead).

Histological and immunohistochemical stainings of the aortic root in LDLR^−/−^ApoB^100/100^ mice and C57BL/6N mice are shown in Figure 1. All LDLR^−/−^ApoB^100/100^ mice showed extensive atherosclerosis in the aorta, whereas C57BL/6N mice had lesion-free arteries. The IMR in the aortic root was 2.0 ± 0.6 for LDLR^−/−^ApoB^100/100^ mice reflecting significant plaque burden. The atherosclerotic plaques in LDLR^−/−^ApoB^100/100^ mice contained various amounts of macrophages (Mac-3 positive staining ranging from 3.7% to 53% of the plaque area). A colocalisation of TSPO staining and Mac-3 staining was detected in the plaque intima (Figures 1(b) and 1(c)). However, TSPO staining was also seen in some Mac-3 negative cells. The medial layer of vessel wall was generally devoid of Mac-3 staining in both LDLR^−/−^ApoB^100/100^ and C57BL/6N mice (Figures 1(b) and 1(e)), whereas TSPO staining in the vessel wall was intense in both strains (Figures 1(c) and 1(f)).

### 3.2. In Vivo PET/CT Imaging

**Figure 2:**
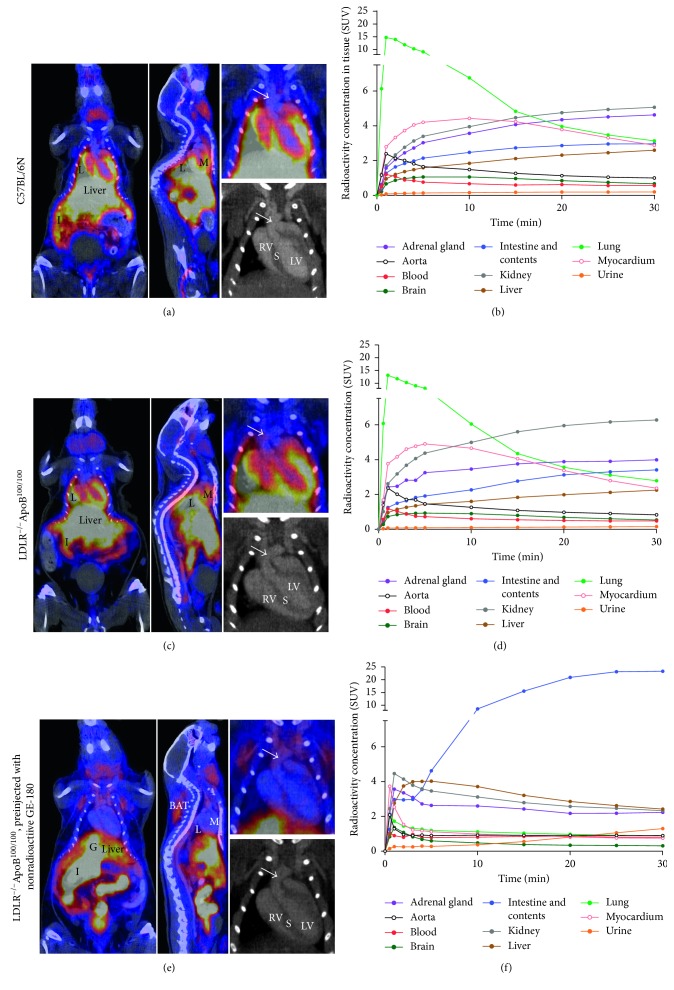
Representative *in vivo* PET/CT images. (a) Coronal PET/CT image shows the sum of ^18^F-GE-180 radioactivity within 0–30 minutes after injection in a healthy C57BL/6N mouse. High radioactivity concentration is seen in the lungs (L), myocardium (M), and in the intestine and its contents (I). Close-up of the thoracic area is shown, with an arrow pointing to the aortic arch. Left and right ventricles as well as septum (LV, RV, and S) are annotated in the CT image. (b) Mean time-activity curves derived from six C57BL/6N mice show rapid peaking in the lung radioactivity concentration. (c) PET/CT in LDLR^–/–^ApoB^100/100^ mice. In the thoracic close-up, the arrow points to a plaque in the aortic arch. (d) Mean time-activity curves derived from six LDLR^–/–^ApoB^100/100^ mice. (e) The effect of preinjection with nonradioactive GE-180 to the ^18^F-GE-180 distribution. Radioactivity concentration is increased in the gallbladder (G), intestine and its contents, and brown adipose tissue (BAT) and decreased in the lungs and myocardium. Close-up of the thoracic area is shown, with an arrow pointing to plaque in the aortic arch. (d) Mean time-activity curves derived from three LDLR^–/–^ApoB^100/100^ mice preinjected with nonradioactive GE-180. Highest radioactivity concentration is observed in the intestine and its contents.

*In vivo* PET/CT imaging showed rapid clearance of ^18^F-GE-180 from the blood circulation and high, transient retention in the lungs. The highest retention was observed at 1-minute time point (SUV 13 ± 2.6), which lowered to SUV 2.8 ± 0.45 at 30 minutes. High retention of radioactivity was seen in the kidneys, adrenal glands, intestine and its contents, and myocardium (Figure 2(a)). Radioactivity concentration in the aorta was similar in LDLR^−/−^ApoB^100/100^ mice and C57BL/6N mice (SUV 0.87 ± 0.18 versus 1.0 ± 0.12). At 20–30 minutes after injection, LDLR^−/−^ApoB^100/100^ mice had significantly lower radioactivity concentration in the brain compared to the C57BL/6N mice (SUV 0.57 ± 0.10 versus 0.71 ± 0.08, *p*=0.028) and higher retention in the kidneys (6.23 ± 0.85 versus 5.01 ± 0.84, *p*=0.038). In the other analysed tissues, no differences between the strains were observed (Table 2). Preinjection of excess nonradioactive GE-180 had no effect on the radioactivity concentration measured in the aorta in LDLR^−/−^ApoB^100/100^ mice (SUV 0.90 ± 0.13). However, it resulted in reduced radioactivity concentration in the adrenal glands, brain, kidneys, lungs, myocardium, and spleen and increased radioactivity concentration in brown and white adipose tissue, blood, intestine and its contents, and urine (Figure 2(b), Table 2).

### 3.3. Ex Vivo Biodistribution

The ex vivo biodistribution of ^18^F-GE-180 at 60 minutes after injection was in line with the results obtained from the PET/CT imaging with few exceptions (Table 3). The radioactivity concentration in the aorta was similar between the LDLR^−/−^ApoB^100/100^ and the C57BL/6N mice (SUV 2.0 ± 0.45 versus 2.5 ± 0.40). Preinjection of nonradioactive GE-180 in LDLR^−/−^ApoB^100/100^ mice had no significant effect (SUV 1.4 ± 0.20). The radioactivity concentration in the intestine was not increased by preinjection of nonradioactive GE-180 in the ex vivo measurements. On the contrary, it caused reduction in the radioactivity concentration in the pancreas in the ex vivo measurements, which was not seen in the PET/CT results.

### 3.4. Autoradiography

**Figure 3:**
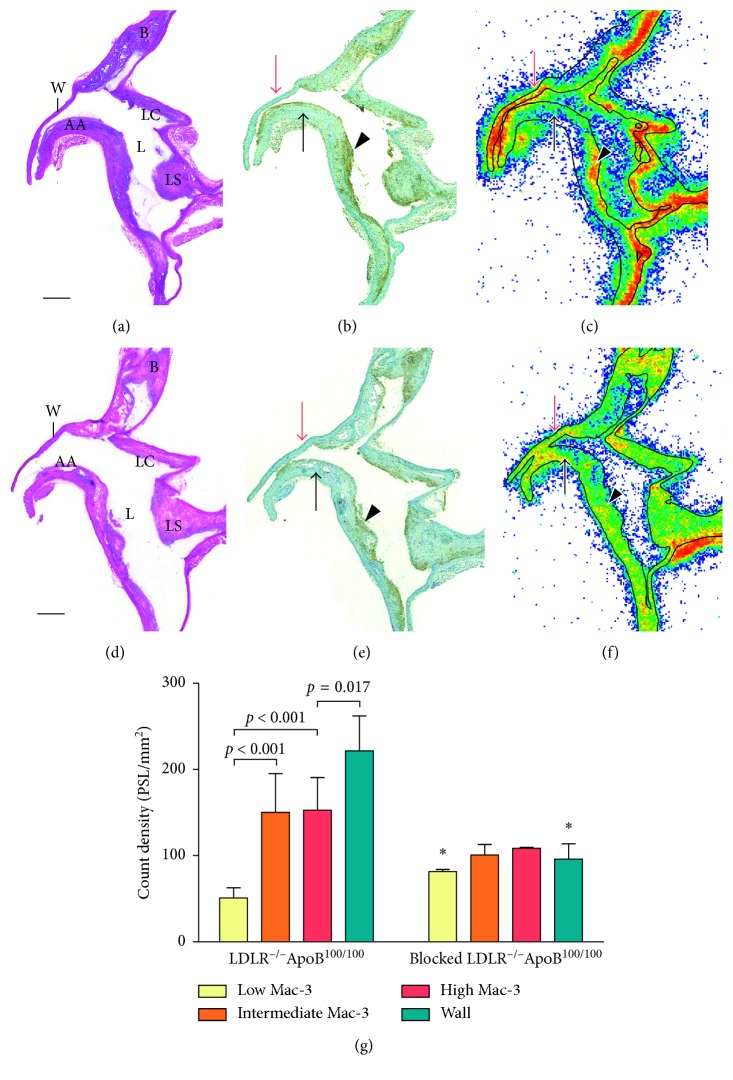
Ex vivo autoradiography of aortic sections in LDLR^–/–^ApoB^100/100^ mice. Longitudinally cut aortic arch stained with hematoxylin-eosin (a, d) shows large atherosclerotic plaques in the inner curvature of the arch and its all branches. Scale bar is 1 mm. AA = aortic arch, B = brachiocephalic trunk, LC = left common carotid artery, LS = left subclavian artery, L = lumen, and W = lesion-free vessel wall. Macrophage immunostainings (b, e) show areas of high (arrowhead) and low (black arrow) macrophage infiltration in the plaques. Lesion-free vessel wall is indicated with a red arrow. (c) Autoradiography shows high ^18^F-GE-180 count density (red colour) in the macrophage-rich areas in plaques as well as in the lesion-free vessel wall. Plaque areas with low macrophage infiltration show low count density (blue to green). (f) Autoradiography in mice preinjected with nonradioactive GE-180 shows similar level of count density in lesion-free vessel wall and all plaque areas. (g) Quantitative results of the autoradiography. ANOVA with Tukey's correction were used in assessing differences between analysed areas within each mouse group. Asterisk indicates statistically significant difference to nonblocked LDLR^−/−^ApoB^100/100^ mice in Student's *t-*test.

In autoradiography images, ^18^F-GE-180 retention in aortic atherosclerotic plaques was assessed in the areas with low, intermediate, and high macrophage densities as well as in lesion-free vessel wall. In the plaques, the count density was most prominent in the areas with high and intermediate macrophage density (150 ± 38 PSL/mm^2^ and 150 ± 45 PSL/mm^2^, resp.) (Figure 3). The percentages of macrophage staining were 33% ± 8.1% and 24% ± 6.0% in those areas. The count density in the areas of low macrophage infiltration (8.9% ± 3.3% macrophages) was significantly lower to both intermediate and high macrophage density areas (51 ± 12 PSL/mm^2^, *p* < 0.001). However, the retention in the vessel wall without lesion formation was even more prominent (220 ± 41 PSL/mm^2^, *p* < 0.001, *p*=0.013, and *p*=0.017 to areas of low, intermediate, and high macrophage density, resp.). Preinjection with nonradioactive GE-180 significantly increased the count density in the areas with lowest macrophage density (81 ± 2.5 PSL/mm^2^, *p*=0.002) and decreased the count density in the lesion-free vessel wall (100 ±18 PSL/mm^2^, *p*=0.002). Preinjection of nonradioactive GE-180 tended to decrease the count density in the plaque areas with highest and intermediate macrophage densities (110 ± 0.93 PSL/mm^2^, *p*=0.091 and 100 ± 12 PSL/mm^2^, *p*=0.11, resp.). In the mice preinjected with nonradioactive GE-180, the percentages of macrophage staining were 43% ± 8.8%, 26% ± 8.8%, and 9.3% ± 4.7% in plaque areas with high, intermediate, and low macrophage infiltration, respectively. Thus, no significant difference in the macrophage percentages between preinjected and the other LDLR^–/–^ApoB^100/100^ mice was observed.

## 4. Discussion

Several different targets for PET imaging of inflammation in atherosclerosis have been studied [21]. As an imaging target for atherosclerosis, TSPO is attractive since it is highly expressed in macrophages [14]. In the PET imaging of vascular inflammation with TSPO-targeting tracers, ^11^C-(*R*)-PK11195 has shown great potential especially in imaging vasculitis [8, 22]. In imaging atherosclerosis, where the inflammatory process is not as prominent, ^11^C-(*R*)-PK11195 has shown lower target-to-background ratio [13]. Novel TSPO-targeting tracers have been studied in preclinical settings to overcome this problem. For example, ^18^F-FEDAA1106 has been evaluated in the imaging of the carotid artery cuff model in mice [23] and ^11^C-PBR28 in a rat model of aortic aneurysm [24]. Both of these studies showed noticeable tracer uptake in lesion areas. However, the models represent an induced, intense local inflammation in the vessel wall, which might not translate to the clinical situation in atherosclerotic patients. The model used in the current study might mimic the clinical atherosclerotic disease better, since there is variable inflammatory activity in the plaques. In previous studies with the same model, both ^11^C-(*R*)-PK11195 [10] and a novel TSPO tracer ^18^F-FEMPA [11] have shown uptake in macrophage-rich plaques, but high tracer retention has been observed in the lesion-free vessel wall.

The third-generation TSPO radioligand ^18^F-GE-180 evaluated in the current study showed similar binding characteristics. It showed uptake in macrophage-rich areas within atherosclerotic lesions as well as in lesion-free vessel wall. Tracer retention was most prominent in tissues with high TSPO expression, such as the lungs and adrenal glands [25]. Preinjection with nonradioactive GE-180 tended to reduce the count density in macrophage-rich plaque areas. In addition, it reduced the retention in tissues with high expression of TSPO, such as the lungs and myocardium, and increased the proportion of tracer remaining in the circulation and taken up in adipose tissue. The kinetics of the tracer were favorable for *in vivo* imaging, since the blood clearance was rapid. However, no differences in aortic radioactivity concentration were observed between atherosclerotic and healthy mice, and the prominent retention in lesion-free vessel wall as well as in the lungs and myocardium might cause limitations.

In addition to high retention in macrophage-rich plaque areas, the ^18^F-GE-180 retention in the vessel wall without lesion formation was prominent. Therefore, the aortic radioactivity concentration, measured both *in vivo* and ex vivo, showed no difference between LDLR^–/–^ApoB^100/100^ and C57BL/6N mice. As shown by immunohistochemistry, macrophages as well as smooth muscle cells and endothelial cells in the aortic wall were positive for TSPO in both mouse strains. High TSPO radioligand uptake to vessel wall has been observed in the same model as in the current study [10, 11] and in a rat model of aortic aneurysm [26]. However, similar vessel wall uptake of TSPO radioligands was not reported in another rat or mouse models [23, 24], nor in the previous studies with ^11^C-(*R*)-PK11195 in patients [13, 22]. The reason for this might be differences in the TSPO expression or differences between the studied radioligands. TSPO is expressed in various cell types of the circulatory system, such as vascular smooth muscle cells and endothelial cells, and its expression shows variation depending on the physiological state [27]. For example, the expression of TSPO in vascular endothelial cells is suggested to be reactive to proinflammatory stimuli [28]. The expression of TSPO in vascular smooth muscle cells also shows variation between species [29, 30]. Despite TSPO is expressed in various cells of vessel wall, its expression in macrophages is significantly higher than in the other cell types present in the vascular wall in humans [14]. Therefore, the observed high retention in lesion-free vessel wall in mice might not preclude the use of ^18^F-GE-180 in vascular imaging in patients. The observed high uptake of TSPO-targeting tracers in the lesion-free vessel wall seen in the current study and previous studies in the same mouse model [10, 11], however, suggest that other animal models should be chosen for future studies with TSPO-targeting tracers.

The aortic ^18^F-GE-180 radioactivity concentration in LDLR^–/–^ApoB^100/100^ mice was not significantly affected by blocking with nonradioactive GE-180; however, the pattern of uptake in the vessel components altered significantly. Without the preinjection, the ^18^F-GE-180 count density was very focused on plaque areas with intermediate or high macrophage infiltration, and the count density was very low in the areas with low number of macrophages. This reflects well the previously observed high ^18^F-GE-180 uptake in microglial cells and low retention in the noninflamed brain areas [31, 32]. Count density in lesion-free vessel wall was also prominent. Preinjection with GE-180 tended to decrease the count density in plaque areas with intermediate and high macrophage infiltration, whereas it actually increased the count density in areas with low macrophage infiltration. This supports the specificity of ^18^F-GE-180 uptake to macrophages in the plaques, since preinjection with nonradioactive GE-180 increased the nonspecific retention in lipid-rich, acellular areas of the plaques. This may be caused by increased availability of circulating tracer, since the ^18^F-GE-180 SUV in blood was higher in the preinjected mice. The retention of ^18^F-GE-180 in lesion-free vessel wall was also significantly lowered by the preinjection, which was expected due to prominent TSPO staining seen in the histology.

The nonradioactive GE-180 was administered in vehicle containing 20% DMSO and 20% ethanol due to limited solubility of the compound. DMSO is known to have anti-inflammatory properties [33]. Therefore, it is possible that the effects of preinjection with nonradioactive GE-180 might also be mediated by the vehicle. The prominent decrease of ^18^F-GE-180 radioactivity concentration in highly TSPO-expressing tissues, such as the adrenal glands, however, suggests occupation of TSPO by nonradioactive GE-180. Additionally, no difference in the amounts of Mac-3 positive macrophages was seen between preinjected and the other mice. Due to the limitation of having Mac-3 as an only marker of inflammatory cells, the potential acute effect of DMSO to the inflammatory phenotype cannot be ruled out. The lack of more detailed analysis of macrophages and other inflammatory cells is acknowledged as a limitation in this study. Notably, the mouse plaque burden was measured from the aortic root, while the radioactivity measurements and autoradiography were performed from thoracic aorta. This limits the available information on the severity of atherosclerosis in the aortic areas studied for radioactivity.

The high and specific lung uptake of TSPO radioligands has been considered as a potential limiting factor in using TSPO as an imaging target in vascular imaging [34]. The lung uptake is most likely caused by resident inflammatory cells with high TSPO expression [25]. In the current study, we observed a high radioactivity concentration in lungs peaking rapidly and decreasing during the 30-minute dynamic *in vivo* PET/CT imaging. The optimal imaging time frame for ^18^F-GE-180 in neuroinflammatory disease imaging was shown to be 90 minutes or more in previous first-in-man-studies [17, 18]. At later time points, the lung uptake may have decreased to such a level that it does not preclude imaging blood vessels in close proximity. In contrast to the tracer retention in the lungs, the myocardial radioactivity concentration did not show clearance during the 30-minute dynamic imaging. This may cause limitations for imaging of the coronary arteries.

The *in vivo* imaging of small targets, especially in rodents, has certain limitations. Analysing small targets, such as the aortic arch or adrenal glands, is subject to partial volume effects, which lead to lower SUVs *in vivo* than ex vivo. Generally, the SUVs measured from the *in vivo* PET/CT images at 20–30 minutes after injection and the ex vivo SUVs measured at 60 minutes after injection were in line. The observed discrepancy in the intestinal radioactivity concentration between *in vivo* and ex vivo SUVs is probably caused by technical reasons, since analysed intestinal uptake *in vivo* contains both the intestinal tissue and its contents, but for the ex vivo measurement, the contents were removed. The preinjection with nonradioactive GE-180 decreased pancreatic radioactivity concentration in the ex vivo measurements but that was not seen in the *in vivo* results. This could be explained by spillover from surrounding intestinal radioactivity in the *in vivo* images, which was high in the GE-180 preinjected mice.

## 5. Conclusion

^18^F-GE-180 showed specific uptake in macrophages not only in atherosclerotic plaques but also in the lesion free vessel wall in mice. Overall, retention in atherosclerotic lesions did not exceed that in lesion-free vessel wall in this model of atherosclerosis. The third-generation TSPO radioligand ^18^F-GE-180 did not show improved characteristics for imaging atherosclerotic plaque inflammation compared to the previously studied TSPO-targeting tracers.

## Figures and Tables

**Table 1:** Characteristics of studied animals.

	LDLR^–/–^ApoB^100/100^	C57BL/6N	LDLR^–/–^ApoB^100/100^, block
Number of mice	6	6	3
Weight (g)	39.5 ± 6.2	34.6 ± 3.6	30.9 ± 6.3
Age (months)	6.8 ± 0.3	5.7 ± 0.5	7.0 ± 0.2
High-fat diet (months)	4.6 ± 0.1	—	5.0 ± 0.2
Injected radioactivity (MBq)	10.0 ± 0.2	10.1 ± 0.5	10.3 ± 0.2

The data are given in mean ± standard deviation. Block: excess amount of nonradioactive GE-180 given intravenously 5 minutes before administration of ^18^F-GE-180.

**Table 2:** ^18^F-GE-180 *in vivo* PET/CT imaging results at 20–30 minutes after injection.

	LDLR^–/–^ApoB^100/100^ (*n*=6)	C57BL/6N (*n*=6)	*p* value	LDLR^–/–^ApoB^100/100^, block (*n*=3)	*p* value
Adrenal gland	3.95 ± 0.71	4.58 ± 0.58		2.21 ± 0.44	0.003
Aorta	0.87 ± 0.18	1.02 ± 0.12		0.90 ± 0.13	
BAT	0.37 ± 0.12	0.37 ± 0.12		1.37 ± 0.29	<0.001
Blood	0.48 ± 0.10	0.56 ± 0.10		0.81 ± 0.16	0.003
Bone	0.56 ± 0.15	0.45 ± 0.11		0.51 ± 0.24	
Brain	0.57 ± 0.10	0.71 ± 0.08	0.028	0.32 ± 0.02	0.002
Intestine and its contents	3.36 ± 0.88	2.96 ± 0.22		23.21 ± 10.85	<0.001
Kidney	6.23 ± 0.85	5.01 ± 0.84	0.038	2.40 ± 0.47	<0.001
Liver	2.19 ± 0.68	2.53 ± 0.32		2.52 ± 0.44	
Lung	2.95 ± 0.54	3.30 ± 0.72		0.91 ± 0.37	0.001
Muscle	0.27 ± 0.13	0.34 ± 0.07		0.41 ± 0.06	
Myocardium	2.58 ± 0.51	3.10 ± 0.46		0.75 ± 0.09	<0.001
Pancreas	1.86 ± 0.48	1.87 ± 0.24		1.90 ± 0.45	
Spleen	3.65 ± 0.65	3.69 ± 0.86		1.44 ± 0.35	0.002
Thymus	1.02 ± 0.28	1.29 ± 0.14		1.02 ± 0.08	
Urine	0.15 ± 0.03	0.19 ± 0.17		1.18 ± 0.47	<0.001
WAT	0.12 ± 0.02	0.15 ± 0.05		0.48 ± 0.41	0.022

The results are expressed as mean standardized uptake values ± standard deviation extracted from the PET/CT images. *p* values are derived from ANOVA with Dunnett's correction, LDLR^–/–^ApoB^100/100^ mice as the control group. Blank cell in column indicates insignificant *p* value (>0.05). Block: excess amount of nonradioactive GE-180 given intravenously 5 minutes before administration of ^18^F-GE-180.

**Table 3:** ^18^F-GE-180 ex vivo biodistribution at 60 minutes after injection.

	LDLR^–/–^ApoB^100/100^ (*n*=6)	C57BL/6N (*n*=6)	*p* value	LDLR^–/–^ApoB^100/100^, block (*n*=3)	*p* value
Adrenal gland	20.79 ± 5.08	33.87 ± 5.24	0.001	4.12 ± 2.12	0.001
Aorta	2.03 ± 0.45	2.52 ± 0.40		1.37 ± 0.20	
BAT	0.84 ± 0.25	0.56 ± 0.18		3.37 ± 0.49	<0.001
Blood	0.23 ± 0.05	0.16 ± 0.02	0.046	0.38 ± 0.08	0.001
Bone	0.99 ± 0.26	0.88 ± 0.18		0.75 ± 0.19	
Brain	0.48 ± 0.05	0.51 ± 0.07		0.31 ± 0.05	0.004
Intestine	6.13 ± 1.44	5.76 ± 1.21		3.14 ± 1.50	0.017
Kidney	15.71 ± 10.27	9.97 ± 3.32		2.53 ± 0.29	0.037
Liver	3.20 ± 0.71	5.14 ± 1.07	0.005	2.50 ± 0.68	
Lung	13.93 ± 9.21	9.85 ± 3.14		2.55 ± 0.74	0.046
Muscle	0.35 ± 0.09	0.38 ± 0.05		0.32 ± 0.02	
Myocardium	2.54 ± 0.47	2.31 ± 0.34		0.71 ± 0.13	<0.001
Pancreas	3.07 ± 0.69	2.46 ± 0.44		0.80 ± 0.11	<0.001
Spleen	6.85 ± 3.44	6.48 ± 2.11		1.94 ± 0.53	0.039
Thymus	1.36 ± 0.39	2.03 ± 0.37	0.011	1.42 ± 0.13	
Urine	0.14 ± 0.06	0.20 ± 0.14		3.61 ± 2.60	0.001
WAT	0.22 ± 0.19	0.14 ± 0.09		0.36 ± 0.09	

The results are expressed as standardized uptake values ± standard deviation. *p* values are derived from ANOVA with Dunnett's correction, LDLR^–/–^ApoB^100/100^ mice as the control group. Blank cell in column indicates insignificant *p* value (>0.05). Block: excess amount of nonradioactive GE-180 given intravenously 5 minutes before administration of ^18^F-GE-180.

## Data Availability

All data supporting the results can be found at Turku PET Centre, Turku, Finland, or from the corresponding author upon request.

## Abbreviations

^18^F-FDG: 2-deoxy-2-[^18^F]-fluoro-*D*-glucose
CT: Computed tomography
DMSO: Dimethyl sulfoxide
^18^F-GE-180: S-*N*,*N*-diethyl-9-[2-^18^F-fluoroethyl]-5-methoxy-2,3,4,9-tetrahydro-1H-carbazole-4-carboxamide
IMR: Intima-to-media ratio
PET: Positron emission tomography
PSL/mm^2^: Photo-stimulated luminescence per square millimeter
ROI: Region of interest
SUV: Standardized uptake value
TSPO: 18 kDa translocator protein.
